# Shaping corticostriatal connectivity with STDP

**DOI:** 10.1186/1471-2202-12-S1-P181

**Published:** 2011-07-18

**Authors:** Jyotika Bahuguna, Man Yi Yim, Ad Aertsen, Arvind Kumar

**Affiliations:** 1 Bernstein Center Freiburg, University of Freiburg, Germany; 2 Neurobiology & Biophysics, Faculty of Biology, University of Freiburg, Germany; 3 Computational Biology, School of Computer Science and Communication, KTH, Stockholm, Sweden

## 

The striatum, the main input nucleus of the basal ganglia, is one of the major sites for learning and decision making. The striatum receives massive convergent inputs from the cortex. Task related cortical activity show weak pairwise correlations, thus, individual medium-sized spiny neurons (MSNs) in the striatum are very likely to receive correlated activity from the cortex. Surprisingly, although cortical neurons outnumber MSNs and each MSN receives around 10,000 cortical inputs, neighboring medium-sized spiny neurons (MSNs) in the striatum are not likely to share their presynaptic inputs [1]. This special arrangement of corticostriatal projections and their inputs is thought to be important to obtain a better signal representation in the striatum [2].

How does this specific anatomical structure of the corticostriatal projection come about? Corticostriatal synapses show a gamut of spike-time-dependent plasticity (STDP) rules which can influence the structure of these projections [3]. Therefore, here, we explored the functional consequences of different STDP rules in shaping the structure of the corticostriatal projections.

Specifically, we studied how the synaptic connections change when a striatal neuron receives a mixture of correlated and uncorrelated synaptic input spikes. Because a neuron is more likely to elicit a spike in response to coincident inputs, the synapses receiving correlated inputs are expected to strengthen over time, whereas those receiving uncorrelated inputs are expected to undergo long-term depression, due to the depression-dominated features of the STDP rule. Furthermore, recurrent inhibition among MSNs implies that STDP would also organize corticostriatal projections such as to reduce the input correlations and sharing of presynaptic neurons among MSNs coupled with recurrent inhibitory synapses. The detailed structure of corticostriatal projections, therefore, depends on the intricate statistics of the cortical inputs, recurrent connectivity and activity within the striatum.

In summary, here we show that standard STDP may explain the observed anatomical structure of the corticostriatal projections. Thus, an interplay of different corticostriatal STDP rules, together with the interactions in the network of MSNs and striatal interneurons, may encode signals from the cortex and modulate the activity of striatal neurons, in particular, the MSNs, which provide the striatal output to the downstream nuclei of the basal ganglia.
